# Application of openEHR archetypes to automate data quality rules for electronic health records: a case study

**DOI:** 10.1186/s12911-021-01481-2

**Published:** 2021-04-03

**Authors:** Qi Tian, Zhexi Han, Ping Yu, Jiye An, Xudong Lu, Huilong Duan

**Affiliations:** 1 College of Biomedical Engineering and Instrument Science, Zhejiang University, Zheda Road 38, Hangzhou, 310027 China; 2 Centre for Digital Transformation, School of Computing and Information Technology, University of Wollongong, Wollongong, NSW 2522 Australia; 3 School of Industrial Engineering, Eindhoven University of Technology, Eindhoven, The Netherlands; 4 Key Laboratory for Biomedical Engineering, Ministry of Education, Hangzhou, China

**Keywords:** Data quality assessment, Data quality rule, OpenEHR archetypes, Automatic, Secondary use of EHR

## Abstract

**Background:**

Ensuring data is of appropriate quality is essential for the secondary use of electronic health records (EHRs) in research and clinical decision support. An effective method of data quality assessment (DQA) is automating data quality rules (DQRs) to replace the time-consuming, labor-intensive manual process of creating DQRs, which is difficult to guarantee standard and comparable DQA results. This paper presents a case study of automatically creating DQRs based on openEHR archetypes in a Chinese hospital to investigate the feasibility and challenges of automating DQA for EHR data.

**Methods:**

The clinical data repository (CDR) of the Shanxi Dayi Hospital is an archetype-based relational database. Four steps are undertaken to automatically create DQRs in this CDR database. First, the keywords and features relevant to DQA of archetypes were identified via mapping them to a well-established DQA framework, Kahn’s DQA framework. Second, the templates of DQRs in correspondence with these identified keywords and features were created in the structured query language (SQL). Third, the quality constraints were retrieved from archetypes. Fourth, these quality constraints were automatically converted to DQRs according to the pre-designed templates and mapping relationships of archetypes and data tables. We utilized the archetypes of the CDR to automatically create DQRs to meet quality requirements of the Chinese Application-Level Ranking Standard for EHR Systems (CARSES) and evaluated their coverage by comparing with expert-created DQRs.

**Results:**

We used 27 archetypes to automatically create 359 DQRs. 319 of them are in agreement with the expert-created DQRs, covering 84.97% (311/366) requirements of the CARSES. The auto-created DQRs had varying levels of coverage of the four quality domains mandated by the CARSES: 100% (45/45) of consistency, 98.11% (208/212) of completeness, 54.02% (57/87) of conformity, and 50% (11/22) of timeliness.

**Conclusion:**

It’s feasible to create DQRs automatically based on openEHR archetypes. This study evaluated the coverage of the auto-created DQRs to a typical DQA task of Chinese hospitals, the CARSES. The challenges of automating DQR creation were identified, such as quality requirements based on semantic, and complex constraints of multiple elements. This research can enlighten the exploration of DQR auto-creation and contribute to the automatic DQA.

**Supplementary Information:**

The online version contains supplementary material available at 10.1186/s12911-021-01481-2.

## Background

With the increasing adoption of electronic health records (EHRs) globally, there is an increasing demand for secondary use of EHR data for research and clinical decision support. The perceived benefits include reducing the cost of data collection, facilitating evidence-based research, and health quality improvement [1–3]. However, concerns about the quality of EHR data have hindered this secondary usage because only when data is of appropriate quality, will their use generate reliable evidence and support research that will lead to clinical outcomes [4]. Therefore, effective data quality assessment (DQA) to ensure adequate data quality is essential for reusing EHR data. This has seen increasing attention to clinical data quality assessment [5].

### Current research about data quality and data quality assessment

Many researchers have investigated the nature and dimensions of data quality, and frameworks and evaluation methods for DQA of EHR data [5, 6]. The recent focus is on frameworks for DQA. The representative work included the data quality ontology developed by Johnson et al. in 2015 [7]; the DQA guideline proposed by Weiskopf et al. in 2017 [8]. In 2016, Kahn et al. proposed a framework to harmonize the existing data quality catalogue into a comprehensive, unified terminology to guide DQA of EHR data (see Table 1) [9]. As a general DQA framework for the secondary use of EHR data, Kahn’s framework summarized classifications of common data quality problems in EHR data. Researchers can define specific quality requirements for data elements based on this framework. It has good coverage to other similar DQA frameworks and is widely adopted by many researchers and organizations for DQA applications [9–12].

**Table 1:** The details of Kahn’s DQA framework

Definition of assessment dimensions	Sub-dimension	Definition
Conformance: whether data value fulfills certain standards and formats	Value conformance: data value conforms to prespecified data types, data domain, allowable values, value sets, or terminology standards	Data values conform to internal formatting constraints
		Data values conform to allowable values or ranges
	Relational conformance: data value conforms to relational constraints imposed by physical database structure	Data values conform to relational constraints
		Unique (key) data values are not duplicated
		Changes to the data model or data model versioning
	Computational conformance: calculated value is consistent with technical functional specification	Computed values conform to computational or programming specifications
Completeness: features that describe the frequencies of data attributes present in a data set without reference to data values	–	The absence of data values at a single moment in time agrees with local or common expectations
		The absence of data values measured over time agrees with local or common expectations
Plausibility: features that describe the believability or truthfulness of data values	Uniqueness plausibility: objects appear multiple times are not duplicate or cannot be distinguished	Data values that identify a single object are not duplicated
	Atemporal plausibility: observed data values, distributions, or densities agree with local or “common” knowledge (Verification) or from comparisons with external sources that are deemed to be trusted or relative gold standards (Validation)	Data values and distributions agree with an internal measurement or local knowledge
		Data values and distributions for independent measurements of the same fact are in agreement
		Logical constraints between values agree with local or common knowledge (includes “expected” missingness)
		Values of repeated measurement of the same fact show expected variability
	Temporal plausibility: time-varying variables change values as expected based on known temporal properties or across one or more external comparators or gold standards	Observed or derived values conform to expected temporal properties
		Sequences of values that represent state transitions conform to expected properties
		Measures of data value density against a time-oriented denominator are expected based on internal knowledge

An effective approach of DQA is to represent data quality requirements in consistency rules [13], e.g. data quality rules (DQRs) or checks [14]. DQRs have been widely used in clinical studies for detecting imperfect data [15–17]. Usually, the creation of DQRs is completed by the evaluator based on the understanding of quality requirements of the use purpose. However, due to the difference in DQA experience of different people, it is difficult to guarantee standard and comparable DQA results in manual-creation of DQRs [5]. An automatic approach helps to fill this gap [7, 18]. In addition, creating DQRs manually to assess the quality of EHR data is time-consuming and labor-intensive, because EHR data usually involves hundreds of data fields. Therefore, researchers have strived to automate the process of DQR creation, which is the challenge for automating the rule-based DQA [19]. Jasna et.al. developed a tool to create DQRs via user-interfaces based on pre-stored rule information for data extraction-transformation-loading (ETL) [20]. However, it is still in the early stage to automatically create DQRs to assess the quality of EHR data [21]. According to Kahn’s DQA frameworks, the creation of DQR not only requires knowledge of data structure but also clinical knowledge [9]. Therefore, a computer-recognizable clinical knowledge model is necessary for automatic DQR creation. Clinical information models (CIM) are formal specifications for representing the structure and semantics of clinical content within health information systems [22]. Such knowledge contained in CIM is computer-readable and available for quality assessment; therefore, it can be useful for automating DQA. However, to the best of our knowledge, to date, there is no published research on this topic. This is a significant research gap that needs to be addressed, giving the importance of reusing EHR data for improving healthcare quality, safety, and efficiency.

### The openEHR and the Shanxi Dayi Hospital

‘OpenEHR’ is an open-source technology for e-health, consisting of specifications, clinical models and software that can be used to create standards, and build information and interoperability solutions for healthcare [23]. The openEHR modeling approach represents clinical information semantics into two levels: the reference model (RM) and the archetype model [24]. The RM defines generic data types, basic structures and features needed to express EHR data instances [25]. The archetype model is computer-readable specifications that describe the constraints of representing clinical information in electronic health systems [24]. The openEHR developed the archetype definition language (ADL) to define and describe clinical concepts in archetypes (see Additional file 1) [26].

The Shanxi Dayi Hospital is a tertiary hospital in Shanxi Province, China. In order to facilitate the secondary use of clinical data, in 2018, the hospital developed a clinical data repository (CDR) system through openEHR approach. 64 archetypes were created to represent the data of the CDR when developing the CDR system [27]. And these archetypes were implemented as a rational database via an archetype relational mapping (ARM) approach [28]. According to the mapping relationships between archetypes and the database, each attribute of archetypes has a corresponding column name and table name of the CDR database.

Since the openEHR archetypes are machine-readable specifications of clinical concepts [29], we developed a case study of automatic DQR creation in Shanxi Dayi Hospital to investigate the feasibility and challenges of automatic DQR creation based on CIM.

## Methods

### Automatic creation of DQRs based on openEHR archetypes

**Fig. 1:**
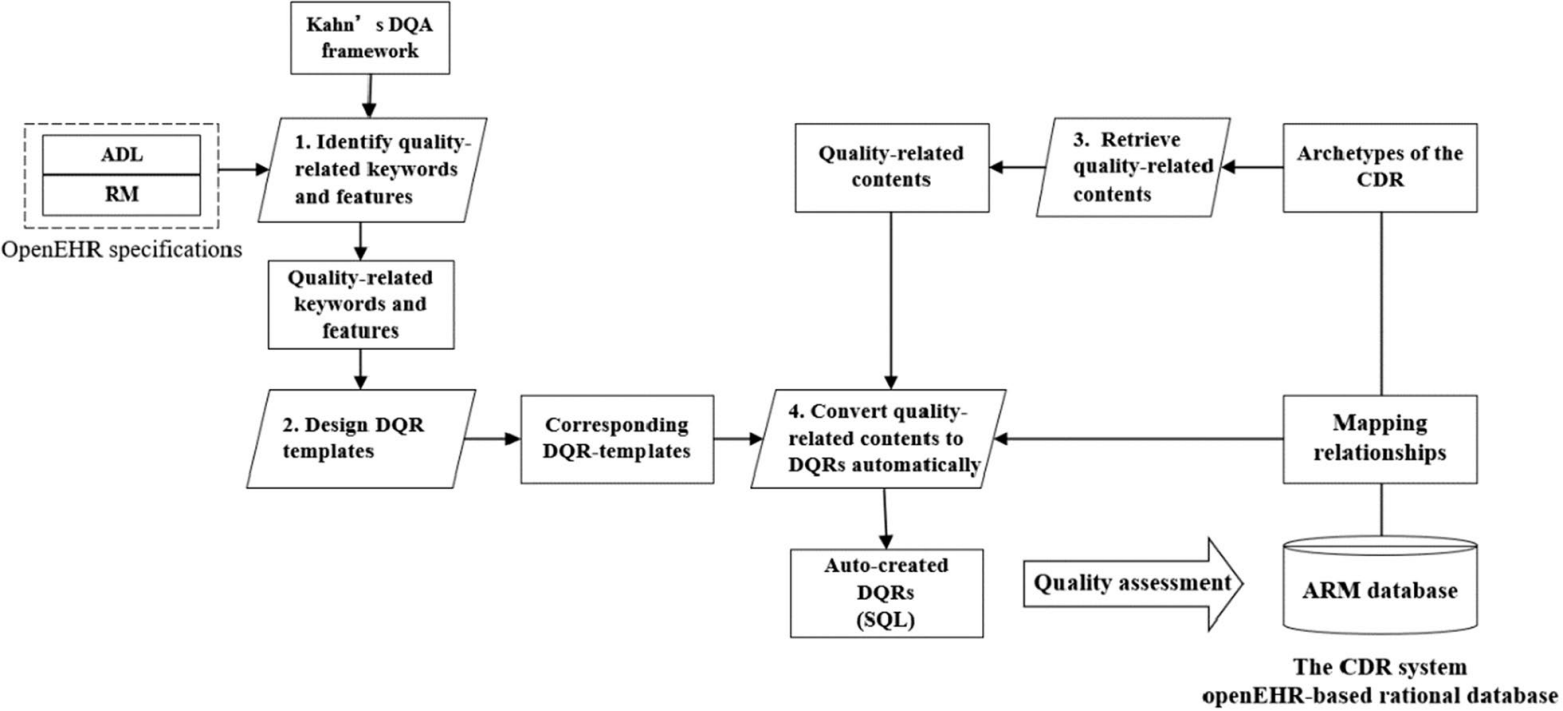
The workflow of automatic creation of DQRs based on the CDR

We developed an approach of automatically creating DQRs based on the ARM database and archetypes. Four steps are involved to retrieve knowledge in archetypes to automatically create DQRs (see Fig. 1).

### Step 1: Identify quality-related keywords and features in openEHR archetypes

We used Kahn’s DQA framework as a reference to identify the keywords and features related to data quality in archetypes. Two specifications of openEHR were used in this process; the specification of openEHR RM and ADL. Three experts (one of archetype, one of DQA, and one biomedical engineer) worked together to map the keywords and their constraint descriptions about data quality in ADL to the sub-dimensions in Kahn’s framework. The mapping criterion is that the keyword or its description has at least one corresponding definition in Kahn’s DQA framework. The RM specification is manually analyzed by the three experts to identify the quality-related features. To ensure that the methods are valid and the results are reliable, a fourth expert in openEHR participated in a meeting to review the results.

### Step 2: Design templates of DQRs based on the identified keywords and features

Each keyword or feature identified in Step 1 expresses one type of quality constraint. These quality constraints were categorized into templates in SQL. Each produced template is an uncompleted SQL-based query that needs to be fed with further information about column information in the “Select” clause, table information in the “From” clause, and constraints information in the “Where” clause (commonly, this clause starts with the word “Where”, but it is not a mandatory word).

### Step 3: Retrieve quality-related contents

The definitions of archetypes are represented in its node/block structure (see Additional file 1). The ADL parser provided by the openEHR Foundation was used to parse the node/block structure into strings of archetype name, node name, keywords, and description of constraints [30]. For example, the constraint “units matches {“mm[Hg]”}”(see Additional file 1: Fig. S1) is parsed as strings: “units”, “matches”, and “mm[Hg]”. There are two types of constraints parsed from the archetypes. The first type is to assign the value or value set of one attribute. It does not have interfering characters, such as “mmHg” or “’Dr’, ‘Miss’, ‘Mrs’, ‘Mr’”, thus can be directly used in DQR templates. The second type includes interfering characters or symbols which are not supported by SQL grammar. For example, “|0.0.1000|” (see Additional file 1: Line 4 in Fig. S1), which means the value range of the attribute should be large than 0 and less than 1000. We extracted “0” and “1000” and converted to “> 0”, “< 1000” and removed the symbol “..”.

### Step 4: Automatic conversion of quality-related contents to data quality rules

According to the mapping relationships of archetypes and database, the parsed archetype name and attribute name were converted to corresponding data table name and column name. Then the data table name and column name, together with the parsed keywords and processed constraints in Step 3 were automatically fed into the corresponding templates of DQRs designed in Step 2 to form the complete SQL queries. This leads to the creation of the corresponding DQRs of the constraints.

### Evaluating the auto-created DQRs

The extent to which the auto-created DQRs can cover actual task requirements is essential for the utility of the automatic approach. To evaluate the utility of the automatic approach, we designed an experiment to evaluate the coverage of auto-created DQRs and actual task requirements.

The Chinese Application-Level Ranking Standard of EHR Systems (CARSES) is the official standard published by the Chinese National Health Commission in 2018 for evaluating and ranking EHR systems in Chinese hospitals [31]. The CARSES specifies the quality requirements of 253 EHR data elements in alignment with the scope of hospital clinical practice. It is a specification of the quality requirements of a typical DQA task in China. It defines data quality requirements of EHR from four dimensions: completeness, consistency, timeliness, and conformity (see Additional file 2). The CARSES is the output of the collaborative efforts of hospital information management experts in China. Its quality requirements of EHR data elements are widely used by Chinese hospitals [31]. Since the CARSES clearly describes the quality requirements of a typical DQA task of EHR data in Chinese hospitals, we designed a three-step experiment based on the CARSES to evaluate the utility of the automatic approach.

### Step 1: Automatic creation of the DQRs based on openEHR archetypes

The clinical data repository (CDR) in the Shanxi Dayi Hospital was built based on 64 openEHR archetypes that represent the data elements of a Chinese EHR system. The CDR contains all the compulsory data elements required by the CARSES. A semantic mapping between data elements of the CARSES and nodes of the 64 archetypes was conducted manually to identify the archetypes that represent the elements of the CARSES. Then these identified archetypes were used to automatically create DQRs following the method developed in the first research component (see Step 3 and Step 4 in Fig. 1).

### Step 2: Experts manual processing to create the “golden standard” DQRs

An expert panel, including one clinical expert and one informatics expert, manually created the DQRs based on the quality requirements of the CARSES and the database structure of the CDR system. An example is shown in Table 2.

**Table 2:** An example of representing a quality requirement of CARSES as an SQL query

Description of quality requirement	Corresponding table name	Corresponding column name	Corresponding DQR(SQL)
ID of request should not be empty	Imaging_exam_ request	Request_identifier	SELECT
			Request_identifier
			FROM
			Imaging_exam_ request
			WHERE
			Request_identifier not null

### Step 3: Evaluate the coverage of auto-created and expert-created DQRs

**Fig. 2:**
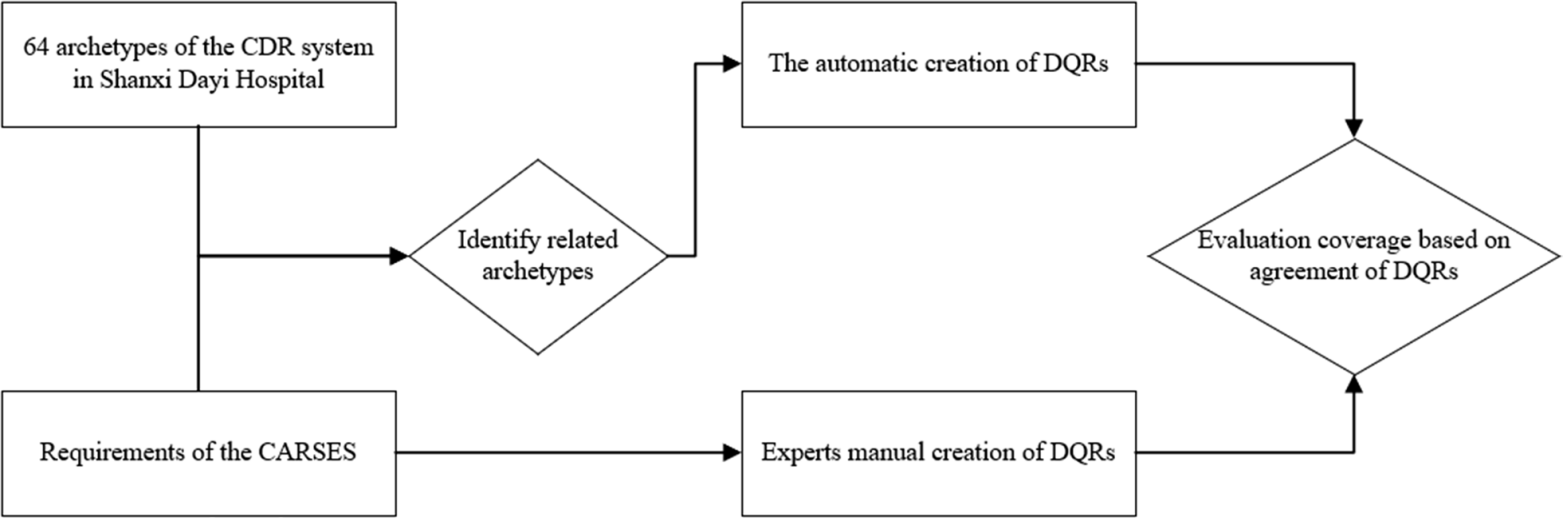
The workflow of verifying the method of automatic creation of DQRs

We compared the expression of the auto-created DQRs and expert-created DQRs to evaluate their level of agreement (see Fig. 2). The evaluation of the agreement based on two aspects: first, we executed auto-created DQRs and expert-DQRs on the same dataset to check whether the results are the same; second, we analyzed the expression of two kinds of DQRs to check whether they are consistent semantically. The auto-created and expert-created DQRs are agreement only if they are the same on both aspects. The coverage is calculated by Eq. (1). The numerator is the number of auto-created DQRs that are in agreement with the expert-created DQRs. The denominator is the total number of expert-created DQRs. We also analyzed the proportion of each dimension of auto-created DQRs and expert-created DQRs.

1 $$Coverage \, = \frac{{the\,number\,of\,auto{\text{-}}created\,DQRs\,which\,are\,in\,agreement\,with\,expert - created\,DQRs}}{total\,of\,expert - created\,DQRs}.$$

## Results

### The quality-related keywords and features in the openEHR Archetypes and their corresponding templates

According to the description of sub-dimensions in Kahn’s framework, we identified five quality-related keywords in openEHR ADL (see Table 3).

**Table 3:** Sub-dimensions in Kahn’s framework and corresponding keywords of ADL and DQR templates

Sub-dimension of Kahn’s DQA framework	Keywords of ADL	Definition	Example of constraint description	DQR templates with example for problematic data (in pseudocode)
Relational conformance	Cardinality	Limits the max number of memberships	(2..5)	Select *
				From archetypes group by attributes having count(*) > 5 and count(*) < 2
	Occurrences	Data exist only once	(1..1)	Select attribute
				From archetype
				group by attribute having count(*) ! = 1
Completeness	Existence	Attribute value is optional	(0..1)	–
		Attribute value is mandatary	(1..1)	Select attribute
				From archetype
				Where attribute is Null or attribute = ‘’
Uniqueness plausibility	Cardinality	Objects in one list are not duplicate	(..unique)	Select *
				From archetypes group by attributes having count(*) > 1
Value conformance	Defining_code	Designate terminology code	(Codeset)	Select attribute
				From archetype
				Where attribute.code not in {Codeset}
	Matches( ∈)	Value range	(|10..1000|)	Select attribute
				From archetype
				Where attribute value < 10 or > 1000
		Designate value	(“mmHg”)	Select attribute
				From archetype
				Where attribute.value ! = ‘mmHg’

The archetype and attribute(s) stand for corresponding table name and column name(s) of the CDR database. Codeset stands for the content of a specific code constraint

One feature of the openEHR RM, the types of Entry archetype, were in accordance with the sub-dimensions in Kahn’s framework (see Table 4). The openEHR RM divides the sequence of a clinical event in five types of Entry archetypes; ADMIN_ENTRY, OBSERVATION, EVALUATION, INSTRUCTION, and ACTION [32]. This feature corresponds to the one definition of temporal plausibility in Kahn’s framework, which requires the data to follow the time logic sequence.

**Table 4:** The quality-related features in the openEHR RM and the corresponding DQR templates.^a^

Sub-dimension of Kahn’s DQA framework	Features	Type	Definition in RM	Use in this study	DQR templates with example for problematic data (in pseudocode)
Temporal plausibility	Instruction, action, observation, evaluation	Entry class	To represent the status of one clinical event	The time sequence logic of one clinical event, for example, the operation request time in Instruction should early than operation executed time in Action	Select attribute
					From archetypes
					Where Instruction. attribute.date/time > action.attribute.date/time

^a^The archetype and attribute stand for corresponding column name and table name of the CDR database

According to these DQRs templates, the quality-related contents retrieved from archetypes are converted to corresponding SQL-based DQRs for assessing data quality. For example, the constraint “magnitude matches {|0.0.1000|}” (see Additional file 1: Fig. S1 line 6) is from the archetype of Blood Pressure. The retrieved quality-related contents are “magnitude”, “matches”, “< 0”, “> 1000”. If the corresponding data table name is blood_pressure, and column name of attribute “magnitude” is diastolic_blood_pressure_value according to the mapping relationships of ARM database. According to corresponding DQR template of Table 3, the auto-created DQRs is “Select diastolic_blood_pressure_value From blood_pressure Where diastolic_blood_pressure_value < 0 and diastolic_blood_pressure_value > 1000”.

### Results of the DQR creation

We mapped the 253 data elements of the CARSES to 27 archetypes in eight categories of healthcare processes: demographic, admission, orders, lab test, imaging examination, electronic medical records (EMR), nursing, and operation. Based on the 27 archetypes, 359 DQRs were automatically created. The experts created 366 DQRs in accordance with the requirements of the CARSES (see Table 5).

**Table 5:** The number of DQRs created by automatic and manual methods using the 27 archetypes

Category	Title of archetype	Auto-created	Expert-created
Demographic	openEHR-DEMOGRAPHIC-PERSON.person	3	2
	openEHR-DEMOGRAPHIC-ITEM_TREE.person_details	7	7
	openEHR-DEMOGRAPHIC-PERSON.person-patient	3	3
	openEHR-DEMOGRAPHIC-PARTY_IDENTITY.person_name	1	1
Admission	openEHR-EHR-ADMIN_ENTRY.admission	7	7
	openEHR-EHR-EVALUATION.problem_diagnosis	6	9
Orders	openEHR-EHR-INSTRUCTION.order	25	20
	openEHR-EHR-ACTION.order	14	14
	openEHR-EHR-INSTRUCTION.prescription	22	22
	openEHR-EHR-ACTION.Prescription	12	12
Lab test	openEHR-EHR-INSTRUCTION.request-lab_test	14	14
	openEHR-EHR-OBSERVATION.lab_test	5	8
	openEHR-EHR-OBSERVATION.lab_test_single	15	17
	openEHR-EHR-CLUSTER.specimen	18	17
Imaging examination	openEHR-EHR-INSTRUCTION.request-imaging_exam	27	28
	openEHR-EHR-OBSERVATION.imaging_exam_image_series	16	17
	openEHR-EHR-OBSERVATION.imaging_exam_report	13	13
EMR	openEHR-EHR- OBSERVATION.EMR_first_page	10	11
	openEHR-EHR- OBSERVATION.EMR_document	11	13
Nursing	openEHR-EHR-INSTRUCTION.nursing	13	15
	openEHR-EHR-ACTION.nursing	14	14
	openEHR-EHR-OBSERVATION.physical_sign	15	15
Operation	openEHR-EHR-INSTRUCTION.request-operation	24	23
	openEHR-EHR-OBSERVATION.operation_record	19	20
	openEHR-EHR-ACTION.operation	12	13
	openEHR-EHR-OBSERVATION.blood_match	15	13
	openEHR-EHR-INSTRUCTION.transfusion	18	18
Sum	27	359	366

### Comparison between the auto-created and the expert-created DQRs

**Fig. 3:**
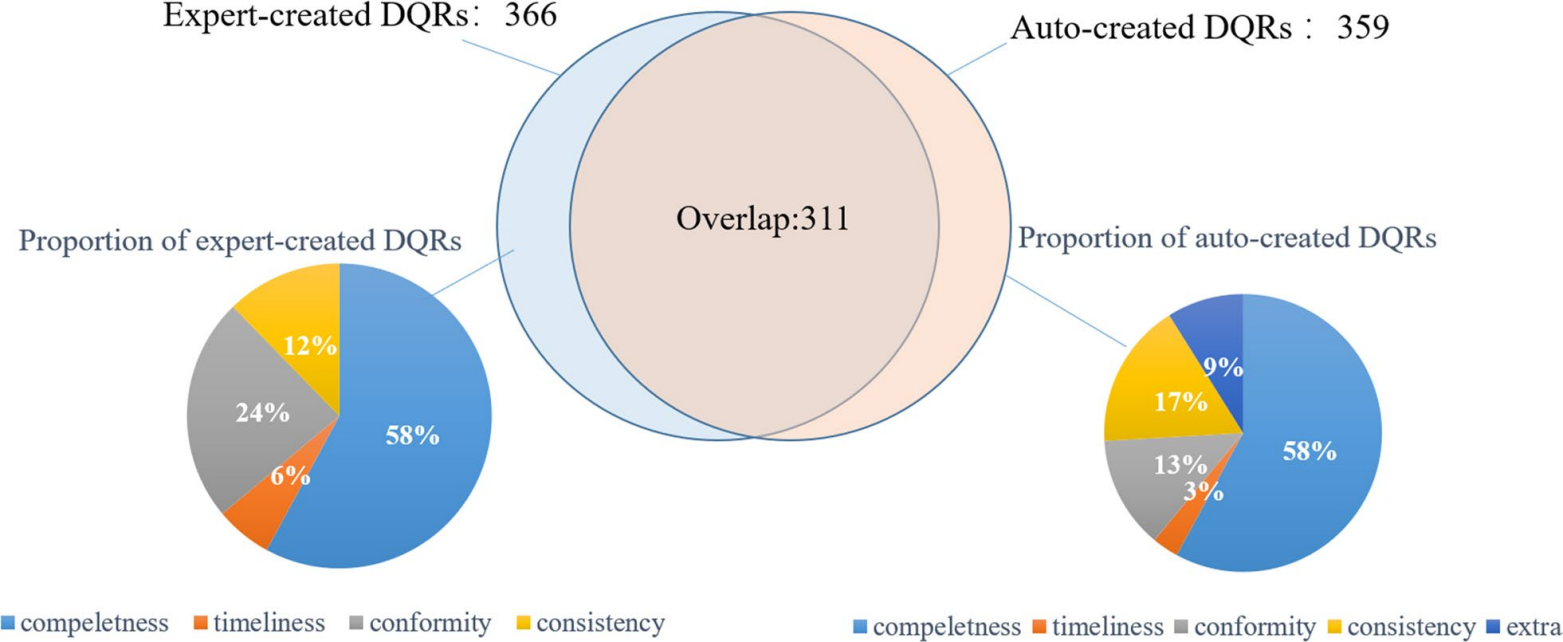
The proportion of expert-created and auto-created DQRs and their coverage

The proportions of the DQRs in each of the four DQR dimensions of the CARSES, completeness, timeliness, conformity and consistency, are shown in Fig. 3. In 359 auto-created DQRs, 208 (57.94%) rules addressed completeness, 47 (13.09%) rules addressed conformity, 61 (17%) rules addressed consistency, and 11 (3.06%) rules addressed timeliness. The rest 32 (8.91%) rules did not match any CARSES dimension.

In 366 expert-created DQRs, 212 (58%) rules addressed completeness, 87 (24%) rules addressed conformity, 45 (12%) rules addressed consistency, and 22 (6%) rules addressed timeliness.

311of the auto-created DQRs were in agreement with the expert-created DQRs; i.e., the auto-created DQRs covered 84.97% of requirements of the CARSES. They included 208 rules of completeness, 45 rules of consistency, 47 rules of conformity, and 11 rules of timeliness. They covered 100% of consistency, 98.11% of completeness, 54.02% of conformity and 50% of timeliness requirements of the CARSES. 16 auto-created DQRs of consistency and 32 auto-created DQRs did not correspond to any expert-created DQRs (see Table 6).

**Table 6:** The number of DQRs and coverage in each dimension of the requirements of CARSES

Category	Completeness		Conformity		Consistency		Timeliness		Extra	Overall	
	Auto	A/E^a^	Auto	A/E	Auto	A/E	Auto	A/E	Auto	Auto	A/E
Demographic	6	6/6	1	1/1	7	6/6	0	0/0	0	14	13/13
Admission	8	8/8	1	1/5	4	2/2	0	0/1	1	14	11/16
Orders	35	35/35	9	9/16	17	13/13	2	2/4	10	75	61/68
Lab test	30	30/30	3	3/13	11	8/8	4	4/5	5	55	47/56
Imaging exam	36	36/37	8	8/12	7	6/6	2	2/3	4	59	54/58
EMR	13	13/16	4	4/6	1	0/0	0	0/2	3	21	17/24
Nursing	25	25/25	8	8/12	5	5/5	2	2/2	2	42	40/44
Operation	55	55/55	15	15/22	9	5/5	1	1/5	7	87	76/87
Sum	208	208/212	47	47/87	61	45/45	11	11/22	32	367	311/366
Coverage (%)		98.11		54.02		100.00		50.00			84.97

^a^Number of agreement rules/ number of expert-created rules

## Discussion

Automatic DQA is significant for facilitating the implementation of DQA and getting standard quality assessment results. Representing data quality requirements as DQRs is the first step towards the automatic DQA of EHR data for secondary use [14, 17]. Many studies have tried to simplify the process of DQR creation. However, the auto-creation of DQRs for DQA of EHR data is still in early-stage [20, 21]. The CIMs provide opportunities to address this methodological gap, yet, to date, there is little research on this front. To explore the feasibility and challenges of auto-creation based on CIM, we developed a case study on a CDR database of a Chinese hospital. In this study, we auto-created DQRs based on openEHR archetypes. It demonstrates that the clinical knowledge embedded in CIMs can be used to automate DQA. The auto-created DQRs are in agreement with 84.97% of the DQRs suggested by the experts for meeting the requirements mandated by the CARSES. Therefore, this study demonstrates that automatic DQR generation based on openEHR archetypes is feasible, and the method is promising for improving the efficiency of DQA.

The auto-created DQRs cover 100% and 98.1% of the requirements in the two dimensions of the CARSES, consistency and completeness, the common requirements for DQA tasks [33–35]. Especially, all the requirements of consistency are covered by the auto-created DQRs, due to the archetype has a good capability of representing code and terminologies constraints. Such constraints are commonly in structured medical data, such as diagnosis, laboratory test, drugs, operations, gender, type of patient, and so on. However, the auto-created DQRs have relatively low coverage of the other two dimensions of data quality in the CARSES, timeliness and conformity, being only 54.02% and 50.00%, respectively. There were no keywords or features of ADL and RM in correspondence with computational conformance and atemporal plausibility of Kahn’s framework. These dimensions are usually relevant to constraints of multiple data elements. In the latest version of ADL, it provides a rules section where researchers can define clinical logic via the openEHR expression language (EL) [26]. The openEHR EL is an expression language developed by openEHR foundation. It defines syntax and grammar to represent clinical logic of clinical elements, such as the calculation of body mass index [36]. The future work can try to utilize all the features of openEHR as much as possible to represent data quality constraints in archetypes.

Three completeness requirements of the CARSES that requires EHR to contain specific information in a plain text, are not covered in the automatic DQRs. For example, CARSES demands the first page of EMR (a plain text) must contain certain specific information. Although, there are only 0.8% (3/366) requirements focus on textual data in the CARSES. Identifying data errors in medical texts is also a need in practice. However, to determine whether a text is error-free, usually need to analyze the semantics of the entities in the text and their relationships, which is difficult to represent as a single logical rule. More advanced algorithms and technologies such as natural language processing (NLP) can be applied for such purposes. It can extract clinical entities and their semantic relationships from a medical text, and further determine whether they conform to corresponding clinical logic.

Indeed, there is a concern about the quality of the auto-created DQRs, as it depends on the quality of the archetypes. Poorly designing of CIMs will lead to lower coverage of auto-created DQRs. However, with the increasing need for standard, shareable clinical data, the CIMs are getting more and more important all over the world. Developing high-quality CIMs before building a clinical information system or application is getting trendy. Creating DQRs automatically based on CIMs is significate for facilitating the implementation of DQA. On the other hand, as a widely spread specification of a CIM, the openEHR has a complete framework of representing clinical information and mature approaches to guarantee researchers can develop high-quality archetypes. Besides, there are already many published studies focusing on developing archetypes accurately [24, 37]. Therefore, in this study, we mainly focused on how to make use of the archetypes in DQA.

There are several limitations to this case study. First, in this study, the CDR database we utilized is an ARM database. The openEHR Foundation does not impose any physical technology for EHR persistence. The auto-created SQL-based DQRs maybe are not implementable in other types of databases. However, no matter what type the database is, as long as there are certain relationships between archetypes and corresponding physical database structure, the methodology is possible to auto-create DQRs for the corresponding database structure. Second, we only applied ADL and RM to discover data-quality related knowledge in archetypes. Future work can explore representing data quality constraints in archetypes with all the features provided by openEHR. Third, to verify mandatory data is not empty, we designed a DQR template to restrict data is null or an empty string. We only considered the common situation in this study. However, in some cases, the empty value may be defined as some other meaningless characters. To facilitate DQA in practice, it is necessary to develop a systematic DQA tool based on this automatic methodology, which assists data managers complete DQRs according to task requirements automatically.

In this study, we investigate the feasibility of creating DQRs based on openEHR archetypes. The processes of this study can also enlighten the studies of automatically creating DQR based on other CIMs, such as HL7 CDA. This study is a preliminary exploration of auto-creation based on openEHR archetypes. The potential and shortcomings of archetypes are identified in this study. These findings are valuable for further research. Future work can further explore data quality constraints in archetypes to enhance the coverage of the auto-created DQRs based on archetypes and developed a systematic tool for practice DQA application.

## Conclusion

Creating DQRs automatically based on openEHR archetypes is feasible. This case study evaluated the coverage of the auto-created DQRs to a typical DQA task of Chinese hospitals, the CARSES. The challenges of automating created DQRs were identified, such as quality requirements based on sematic, and complex constraints of multiple data elements. This research contributes to the automatic creation of DQRs. Studies focus on exploring other CIMs for automating DQR creation can be enlightened by this case. Future research can further explore data quality constraints in archetypes to enhance the coverage of the auto-created DQRs.

## Supplementary Information

**Additional file 1.** The illustration of openEHR ADL from openEHR online specification.

**Additional file 2.** An exemplary form of the CARSES.

## Data Availability

Data sharing is not applicable to this article as no datasets were generated or analyzed during the current study.

## Abbreviations

ADL: Archetype definition language
ARM: Archetype relational mapping
CARSES: Chinese Application-Level Ranking Standard of EHR System
CDR: Clinical data repository
DQA: Data quality assessment
DQRs: Data quality rules
EHR: Electronic health record
EMR: Electronic medical record
NLP: Natural language processing
RM: Reference model
SQL: Structured query language
