# Supplementary material for: Application of openEHR archetypes to automate data quality rules for electronic health records: a case study
Source: BMC Med Inform Decis Mak. 2021 Apr 3;21:113. doi: 10.1186/s12911-021-01481-2 (PMC8019503; doi:10.1186/s12911-021-01481-2)
Supplement: Supplementary file 1 — Additional file 1. The illustration of openEHR ADL from openEHR online specification. [file 12911_2021_1481_MOESM1_ESM.docx]

## Additional file 1

**The illustration of openEHR ADL from openEHR online specification**

The archetype definition language (ADL) defines various keywords to describe the diverse constraints of data elements in an archetype. The structure of the archetypes that are used in this research is illustrated in a segment of the ADL in Fig.1. In this archetype, data elements and their constraints are described in block/node structure. Each element is assigned a unique node code (e.g., ‘id10’ in Fig.1 Line 1) and the constraints for this element are described in the blocks in Lines 2 to 9. Usually, one constraint consists of an attribute, a keyword, and constraint for the attribute. The constraint can be a default value, value range, or cardinality of an attribute. In this example, ‘matches’ that appears six times in Lines 1 to 6 is a keyword. The word before it is an attribute, e.g., value, magnitude, property, units. Description of the constraint is presented inside the curly brackets that follow the keyword. For example, in the constraint in Line 6 “units matches {“mm[Hg]”}”(see Fig.1 Line 6), keyword “matches” assigns the default value “mm[Hg]” as a constraint to the attribute “units”.


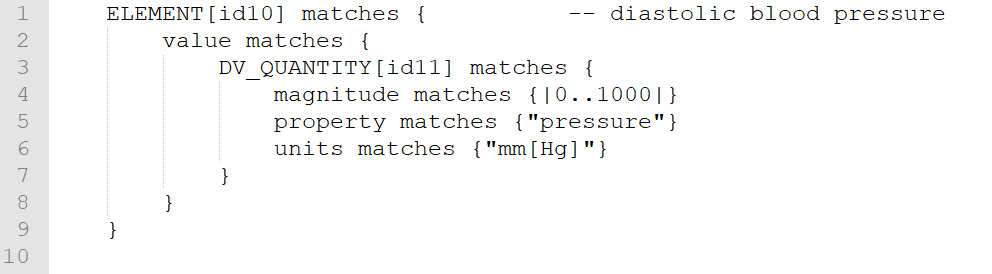


Fig.1 Illustration of the segment of an archetype, adopted from ADL online specification
