# Supplementary material for: Application of openEHR archetypes to automate data quality rules for electronic health records: a case study
Source: BMC Med Inform Decis Mak. 2021 Apr 3;21:113. doi: 10.1186/s12911-021-01481-2 (PMC8019503; doi:10.1186/s12911-021-01481-2)
Supplement: Supplementary file 2 — Additional file 2. An exemplary form of the CARSES. [file 12911_2021_1481_MOESM2_ESM.docx]

## Additional file 2

**Table 1** The English version of an exemplary form of the CARSES

| Item Code | Item of Healthcare Process | Data Item for Quality Assessment. |
| --- | --- | --- |
| 04.01.3 | Request and reservation | **Consistency**: Examination of request records (name of examination, code of examination, and body site of examination should be consistent) |
| 04.01.4 | Request and reservation | **Completeness**:   1. Examination of request records (ID of request, patient ID, patient name, examination item, body site of examination, intent of examination, doctor of request, and department of request should not be empty) 2. Examination reservation records (ID of request, patient ID, patient name, examination item, body site of examination, and arrangement date/time of examination should not be empty) |
| 04.01.5 | Request and reservation | **Completeness**: examination request records (diagnosis, description of special situation, department of execution, location of department, and date/time of request should not be empty)  **Conformity**: examination request records of execution department and examination records of clinical department (ID of request, patient ID, examination item, body site of examination, doctor of request, department of request) should be consistent. |
| 04.01.6 | Request and reservation | **Timeliness**: date/time of request in examination request records early than arrangement date/time of examination in examination reservation records |

*Completeness requires the value of a data element not to be empty. Consistency requires data to be consistent with its corresponding code. Timeliness requires the sequence of data to follow the logic flow of time sequence; for example, date/time of admission must happen before date/time of discharge. Conformity requires the data that present the same thing in different records to be the same; for example, ‘breast cancer surgery’ should be recorded as such in the data record of surgery request, as well as that of surgery procedure. Only after fulfilling these quality requirements, will data be qualified.

**Table 2** The original Chinese version of Table 1

| 项目代码 | 业务项目 | 数据考察项 |
| --- | --- | --- |
| 04.01.3 | 申请与预约 | **一致性**：检查申请记录（检查项目名称、检查项目代码、检查部位） |
| 04.01.4 | 申请与预约 | **完整性**：  1、检查申请记录（申请单编号、病人标识、病人姓名、检查项目、部位、检查目的、申请医师、申请科室）  2、检查预约记录（申请单编号、病人标识、病人姓名、检查项目、部位、检查安排时间） |
| 04.01.5 | 申请与预约 | **完整性**：检查申请记录（诊断、特殊情况描述、执行科室、检查科室位置、申请时间）  **整合性**：检查科室接收的检查申请记录与临床科室的检查检查记录（申请单编号、病人标识、检查项目、部位、申请医师、申请科室）可对照 |
| 04.01.6 | 申请与预约 | **及时性**：检查申请记录（检查申请时间）≤检查预约记录（检查安排时间） |
